#  Knowledge, Attitude and Practice of Pharmacists regarding Dietary Supplements : A Community Pharmacy- based survey in Tehran

**Published:** 2014

**Authors:** Gholamhossein Mehralian, Nazila Yousefi, Farshad Hashemian, Hanieh Maleksabet

**Affiliations:** a *Department of Pharmacoeconomics and Pharma Management, School of Pharmacy, Shahid Beheshti University of Medical Sciences, Tehran, Iran. *; b *School of Pharmacy, Azad University, Tehran, Iran.*

**Keywords:** Dietary supplement, Food Supplementation, KAP study, Community pharmacist, Iran

## Abstract

The present study aimed to evaluate pharmacy practice regarding dietary supplements in Tehran (I.R. Iran). So, the factors affecting on pharmacists' practice including their knowledge, attitude, and some underlying factors were evaluated.

This is an observational knowledge; attitude and practice (KAP) study. The unit of analysis include pharmacies practice located in Tehran. The data was collected in 2013 via an anonymous, self-administered; postal questionnaire consisted of demographic information, knowledge (subjective and objective questions), attitude, and practice evaluation part. Descriptive and inferential statistics were performed using SPSS.

This study showed that although the knowledge has a significant effect on attitude and practice, the attention should be paid on other underlying factors such as experience, pharmacy ownership situation and academic degree which might have positive impact on pharmacists' practice.

According to this study, although many underlying factors such as experience, university and pharmacy ownership have impact on pharmacy practice regarding dietary supplements, the most attention should paid to knowledge as the main factor and more attention should be paid to training on dietary supplement could be recommended.

## Introduction

Following improvement in health expectation of communities, more significant attention is attracted to complementary medicine (1). Dietary supplements (DS) are the most common subset of complementary and alternative medicines (CAM), a group of diverse medical and health care products that are not usually considered as a part of conventional medicines (2).

Following the increasing community awareness regarding the concern of prevention and treatment of diseases, the average consumption of CAMs has been dramatically increased, (3) so that in 2007, about 40% of adults in United States used some form of CAM (4).

The popularity and use of dietary supplements (DS) increased in many developed countries (5). According to a survey conducted In Australia, 52% of the Australian population had used at least one non-physician-prescribed dietary supplement in 2003 that increased to 68.9% in 2007 (6,7). This was 71% in Canadian population in 2005(8). Dietary Supplement market is also increasingly growing in developing countries, so that in Iran the main product in the pharmaceutical counterfeit market was supplements (64.5%) (9) and according to "Research and Market" report on vitamins and dietary supplement in Iran, utilization of dietary supplements in Iran is significant and grows quickly (10). As the consumers spend significant time and money on these products, the safe and effective use of these products could guarantee an appropriate health promotion.

Pharmacists can be consider as the most important resources in health system and may have a great impact on the public health (11). Community pharmacists play an important role in patient training. However, studies show deficiencies in pharmacists-patients communication regarding health products (12, 13) and their need for training to enable them to provide consulting service more effectively (14). 

Most consumers of DS presume the role of pharmacist include recommendation of effective DS products. However, the professional responsibilities of pharmacists with respect to these products have not been well distinguished and pharmacists do not play their professional role properly (15). Moreover, by increase the use of dietary supplements, the number of customers who ask pharmacists about these products has increased (16). In addition, due to nature of the therapeutic conditions, success of complementary medicines highly depends on finding the right therapy which may be different for each individual (17). This motivated us to use knowledge, attitude and practice (KAP) study which assess knowledge, attitude and practice in three different sections and evaluate their correlation in Iranian pharmacist population. Prior studies (18-20) show that consumers routinely ask their pharmacist for information about these products and seek for advice about DS as part of pharmacy practice. Recent survey of pharmacy customers in Australia shows that 87% of consumers expect the pharmacist to be able to provide them enough information about CMs efficacy, and 92% expect to receive safety information regarding these products. Most DS consumers believe that the role of pharmacist should contain recommendations of effective DS products (20). In addition, pharmacists’ reliability to detect and restrain interactions between CMs and conventional medications has been recognized in literature as an important issue (21).

Despite these expectations, pharmacists mostly rate their own knowledge about CAMs as inadequate, and do not feel confident in answering patient's questions, and several studies have illustrated that, pharmacists' lack of knowledge or inadequate education on supplement products incapacitate them to provide informative consultation(16, 22-26), lack of confidence in introducing these products to their customers (27) and, lack of skill (28).

As, to the best of our knowledge there is no study considering pharmacists knowledge, attitude and practice regarding dietary supplements in developing country context. We choosed knowledge, attitude and practice (KAP) study to investigate this problem. Knowledge means understandings but it does not necessarily lead to required behaviour. Attitude is a way of being; a position which is not as directly observable as practices and practice is the observable behaviours of an individual (29). 

## Method

The purpose of the present study is to assess the knowledge, and measure the professional attitude and practice of pharmacists about dietary supplements. KAP study is applied for this study as the most popular and widely used method to assess the knowledge, attitude and practice. KAP survey may study the subjects which could be affected by social, cultural and economic factors, and may influence on the implementation of public health objectives. This is a cross-sectional survey of KAP and the data was gathered in 2013. As usually at least one pharmacist must work at each pharmacy, the number of community pharmacies is considered as our target population. 

The questionnaire was targeted to pharmacists, and also pharmacy students who had passed at least four years at university and work as the temporary pharmacist in community pharmacies. Data was collected via an anonymous, self-administered postal questionnaire. The questionnaires were posted to all 2000 pharmacies in Tehran in September 2013. Comprehensive description about the study was introduced to participants at first page of questionnaire. They were allowed rejecting to answer at the first or withdrawing at any time after. They assured the answers keep confidentially and the names are not disclosed during the study and in reporting final results. 

The questionnaire was developed based on the previous literature (1-3, 30-33) to collect information about pharmacists' knowledge, attitude and practice toward CAMs and dietary supplements. The questionnaire consisted of demographic information, knowledge (subjective and objective questions), attitude, and practice evaluation part. It was validated (face validity and content validity) by ten experts in pharmacy practice area through questionnaire survey. The reliability of questionnaire was tested through analysis methods. 

Demographic section of the questionnaire was designed to collect respondents’ demographic (gender and age) and other background data (years of experience and educational background). Pharmacists were classified to 3 different categories based on financial relation with pharmacy. Next section investigated the pharmacists’ knowledge about dietary supplements (DS) while pharmacists were asked to rate their knowledge mostly based on their perception about their information about safety, adverse effects, and drug interactions of DS. To find out pharmacists’ general attitudes towards DS, the pharmacists were asked to respond to 9 statements pointed to believe and attitude which is mentioned in Table 2. To figure out practice, pharmacists were asked to rate the frequencies of 11 common practice activities which they are required to do (32). Finally, some multi choice questions about indication and contraindications of different dietary supplements were asked from pharmacists to objectively measure their knowledge. 

## Results

Out of 2000 distributed questionnaires, 500 questionnaires were returned. The response rate was 25% and response bias is not considered noting variety of demographic characterization of respondents as shown in Table 1. 

**Table 1:** Demographic and Background Characteristics of participants (N=500).

**Characteristics**	**Categories**	**Number (%)**
**Gender**	Male	329 (65.8)
	Female	171 (34.2)
**Age **(years)	Under 25	165 (33.0)
	25-35	186 (37.2)
	36-45	89 (17.8)
	Above 45	60 (12.0)
**Education**	Pharmacy student	102 (20.4)
	Pharm.D	374 (74.8)
	PhD	24 (4.8)
**Experience in pharmacy practice **(years)	Less than 5	251 (50.2)
	5-10	122 (24.4)
	11-20	78 (15.6)
	More than 20	49 (9.8)
**Position** ** in the pharmacy**	Temporary pharmacist	186 (37.2)
	Technical responsible	216 (43.2)
	Owner	98 (19.6)

Table 2 shows descriptive and reliability analysis. As seen, overall reliability of questions according to Cronbach's alpha reaches to 0.83 which indicate an acceptable reliability. Moreover, descriptive parameters including mean and standard deviation (SD) corresponding to each question and Cronbach's alpha corresponding to each dimensions (knowledge, attitude and practice) are provided. In this table knowledge evaluation is subjective and as it mentioned earlier this is also evaluated objectively by other part of questionnaire which is mentioned in Table 4.

**Table 2:** Descriptive and reliability analysis of KAP questionnaire

**Questions**	**Mean**	**SD**	**Cronbach’s alpha**
**Knowledge (subjective approach)**			
Generally I have sufficient information toward dietary supplement.	3.46	0.73	0.86
I have sufficient information about efficiency and effectiveness of dietary supplement.	2.89	0.87	
I have sufficient information about adverse effects of dietary supplement.	3.32	0.77	
I have sufficient information about dosage and administration of dietary supplement.	2.96	0.81	
I have sufficient information about indications of some dietary supplement in specific groups such as pregnancy, breast feeding, pediatric and geriatric.	3.25	0.77	
I have sufficient information about drug-supplement interactions.	2.99	0.86	
I have sufficient information about contraindication of dietary supplement and in special groups of patient for example with hypertension or kidney disease.	2.85	0.79	
**Attitude**			
Dietary supplements have a positive impact on public health.	2.96	0.87	0.61
Therapeutic efficacy of dietary supplement may be considerable.	3.63	0.78	
Pharmacists should be knowledgeable about supplements and consulting in this field is part of pharmacist’s duties.	3.31	0.88	
	4.11	0.72	
Supplement should dispense according to the nutritionist or physicians prescription.	2.25	0.90	
Supplement should be sold in pharmacies under pharmacist’s supervision.	3.03	0.87	
Supplements considered as an important source of profit for pharmacies.	3.89	0.87	
Price is important factor for recommending supplements to customers.	3.29	0.96	
Customers usually are influenced by Pharmacist’s comments about supplements.	3.68	0.90	
**Practice**			
I always allot enough time for giving advice to customers on supplements.	3.91	0.77	0.81
I’ve studied some scientific references regarding to supplements.	3.60	0.75	
I could refer to valid Web Pages and scientific references relevant to dietary supplement in case of needed.	3.16	0.81	
I always recommend supplements to consumers with confidence about their effectiveness.	3.27	0.86	
I always inform consumers about possible adverse effects of dietary supplements.	3.41	0.85	
I always advise consumers about dosage and administration of supplements.	3.44	0.82	
I always ask consumer’s medical history when I recommend these products.	3.57	0.75	
I always check whether particular supplement taken by consumer interact with her/his prescription medicines.	3.56	0.77	
I always inform consumers about drug-supplement interactions.	3.50	0.84	
I have self-confidence for recommending supplement.	3.43	0.87	

KAP: knowledge, attitude, practice; SD: standard deviation

In Table 3, the factor analysis shows that the KMO (Kaiser-Mayer-Olkin) values were 0.89, 0.56 and 0.87 for knowledge, attitude and practice respectively, which show statistical capability for further reduction.

Besides exploratory factor analysis, to understand the relationship between the observed and latent factors, and to test uni-dimensionality of dimensions, confirmatory factor analysis has been employed. Extracted variables showed that all 3 components have contributed to nearly 60% of the total variance, whereas average variance extracted regarding each component of KAP is above 50% indicating high uni-dimensionality according to Table 3.

**Table 3:** Descriptive, correlation and factor analysis

**Dimensions**	**Mean**	**SD**	**KMO**	**AVE**	**Factor loading**	**Knowledge**	**Attitude**	**Practice**
**Knowledge**	3.10	0.59	0.89	0.55	0.65-0.80	1	0.29**	0.43**
**Attitude**	3.33	0.34	0.56	0.63	0.53-0.70	0.29**	1	0.31**
**Practice**	3.51	0.60	0.87	0.61	0.64-0.74	0.43**	0.31**	1

SD: standard deviation; KMO: Kaiser-Mayer-Olkin; AVE: Average Variance Extracted

** Correlation is significant at the 0.01 level (2-tailed).

As shown in Table 3, correlation analysis shows that there are positive and significant relationships between three components of KAP variable, so that the impact of knowledge on practice is more than on attitude. Furthermore, result of correlation shows that attitude has a positive and significant effect on practice. However, the correlation coefficients in aforementioned test do not show a strong relationship between studied variables. 

As mentioned earlier, in this study to test the knowledge of participants about dietary supplement, besides subjective questions mentioned in section 1 of questionnaire, seven practical questions regarding some important indication and contraindication of some dietary supplements which were proposed by relevant experts have been used. To categorize the practical knowledge of attendees, the score of respondents (mean ± SD: 1.542 ± 0.919) was categorized based upon expert’s opinion accordingly; which above 70% was considered excellent, 60-70% good, 40-60% average and less than 40% weak and poor. As mentioned in Table 4, results show that around 62% of respondent has week practical knowledge, 21% of our sample has average level of practical knowledge and around 17% of participants could get good and excellent score. In addition, there are not any significant differences in scores of different groups of gender, age, experience, educational background.

**Table 4:** Descriptive results of objective practical questions about pharmacists' knowledge (N=500).

**Scoring category**	**Number (%)**
**Poor**	23 (4.6)
**Weak**	288 (57.6)
**Average**	105 (21.0)
**Good**	63 (12.6)
**Excellent**	21 (4.2)

To test the relationship among demographic profile of participants with three components of KAP variables, Kruskal-Wallis as a common approach of non-parametric analysis for nominal variables has been used. As shown in Table 5, there is not any significant difference between genders in attitude and practice. Conversely, the variable of knowledge which subjectively evaluated shows a significant difference with gender item. So, men participants are more likely knowledgeable than women participants according to dietary supplements, and it can be explained that men participants may have more self- confidence. Considering different age and experience groups, there are not any significant differences in attitude; conversely knowledge is growing by age and experience. More interestingly, practice score is growing up to 45 years old and 20 years of experience, and then decline.

Moreover, there is a positive relationship between education level and all component of KAP, so that participants with higher academic degree have, the more knowledge, attitude and practice in dietary supplements have, and it also can be explained same to gender variable. Given to university, pharmacists who graduated abroad show higher level of knowledge or more self- confidence about their knowledge, while attitude and practice do not show any significant differences. 

Related to ownership variable, it has significant and positive effect on knowledge, attitude and practice of pharmacist. 

**Table 5:** Participants’ characteristics and KAP components

**Grouping Variable**		**Test Variables Mean Rank**			
		**Knowledge**	**Attitude**	**Practice**	**Knowledge test Score**
**Gender**	Female	230.41	248.69	249.42	256.67
	Male	289.16	253.99	252.58	238.64
	Sig. (2-tailed)	.000	0.696	0.815	0.138
**Age **Categories (Years)	Under 25	237.57	214.11	187.98	248.15
	25-35	254.07	256.72	274.35	250.11
	35-45	267.08	286.23	281.97	269.59
	Over 45	250.39	278.30	301.84	229.86
	Sig. (2-tailed)	0.452	0.000	0.000	0.316
**Experience Categories **(Years)	Less than 5	238.84	220.62	211.30	242.86
	5-10	272.92	278.23	293.08	254.01
	11-20	260.49	285.19	286.19	279.49
	More than 20	238.52	279.30	288.44	234.74
	Sig. (2-tailed)	0.149	0.000	0.000	0.131
**Education level**	Student	156.48	218.94	198.47	251.09
	Pharm.D	273.34	256.24	260.96	248.43
	PhD	294.25	295.19	308.65	280.27
	Sig. (2-tailed)	0.000	0.020	0.000	0.503
**University location**	Non-governmental	222.59	252.82	239.58	254.91
	governmental	270.76	248.18	257.99	246.49
	Abroad	447.50	416.50	452.50	448.00
	Sig. (2-tailed)	0.000	0.481	0.138	0.238
**Ownership Position**	Duty of director	184.94	222.92	208.87	247.42
	Technical responsible	280.05	261.25	262.78	246.02
	Owner	309.80	279.16	302.44	266.23
	Sig. (2-tailed)	0.000	0.003	0.000	0.402

Descriptive and inferential statistics were calculated using the Statistical Package for Social Sciences (SPSS) version 19.

## Discussion

Following improvement in the quality of life and people heath expectation, the market of dietary supplements is dramatically grown. As it leads to significant expenditure in health system, its public heath impact should be considered. Community pharmacies as the important part of health system providing health services for public health are in the best position to consult customers with evidence-based information about dietary supplements. 

However, according to the previous studies, customers usually are not satisfied by the information received from pharmacists, and also pharmacists are not confident enough to provide such information to their customers (23-25, 27, 34). The previous studies explored that the lack of some factors such as information and understanding of the advantages of DS for general health maintenance, prophylaxis of disease and remedy of minor conditions, knowledge of specific DS products and profile of the DS’ company, may raise customers unsatisfactory (17, 29-31). The most significant pharmacists' barrier to communicate on DS is lack of scientific evidence availability, and also lack of training, lack of accurate and accessible information, lack of reimbursement, legal concerns, time constraints; whereas suggestion of DS products by pharmacies’ staff can be added to aforementioned barrier (9, 23, 38, 39). Proper use of DS products not only can positively impact public health, but also may enhance the esteem of the pharmacy. Therefore, it is considerable to fulfill the consumer’s expectations through the pharmacist–consumer relationship, and more attention should be paid to professional responsibilities of pharmacists about dietary supplements especially in developing countries (8, 23, 40). Moreover, effective pharmacist communication with patients resulted in economic and humanistic outcomes (41). 

To figure out the main factors which affect the final practice of pharmacists on DS, we choose KAP study to discover gaps to propose proper intervention. Comply with expectation; there is a positive and significant relationship between three components of KAP variable. It implies higher knowledge participants have, the more likely they have better attitude and practice. But the correlation coefficients don't show strong relationship between KAP components, so that attitude and practice not only are mainly influenced by knowledge but also are influenced by context, environment and other underlying factors such as pharmacy ownership. In addition, the impact of knowledge on practice is more than on attitude. Related to correlation analysis, results show that attitude has a positive and significant effect on practice, so it is important to find out the attitude influencers to improve practice of pharmacists.

Results of objective knowledge evaluation show that more than half of respondents have a week knowledge without any significant differences in scores of different groups of gender, age, experience, education and university. It shows that more attention should be paid to the educational material in university and continuous training courses. 

Considering demographic profile of participants with three components of KAP variables, some interesting results were provided. Although, men participants are more likely knowledgeable than women participants about dietary supplements, the results does not show any significant differences between genders in attitude and practice. This implies that attitude and practice might be influenced by some other underlying factors like age, experience, education and ownership situation. 

Ownership variable has a significant and positive effect on knowledge, attitude and practice of pharmacists, so that pharmacy owners not only show higher attempt on practice, but also show higher level of knowledge and attitude.

Given to age and experience groups, although knowledge is growing by age and experience, practice score is growing up to age 45 years old and 20 years’ experience and then decline. The positive relationship between education level and all component of KAP show that education not only has an indirect effect on practice through knowledge improvement, but also has direct effect on attitude and practice of pharmacists. So, the results strongly recommend higher education for better practice. 

Given to location of university, pharmacists graduated abroad caught higher score in knowledge, because they might be due to the different training schedule or might be due to more self confidence. However, attitude and practice do not show any significant differences with university location. This result again emphasis that, although the higher knowledge results to higher attitude and practice, it is not the only effective factor and other factors should be considered.

This study strongly recommends higher education and more structured training schedule for pharmacists. In addition, attention should be paid to experience as an important factor. Moreover, the results recommend ownership factor as an important incentive can increase practice of pharmacists regarding dietary supplement. As usually this kind of products have high benefit margin, the owner pharmacists are more interested to learn more about them to sell more. 

Actually the best way for practice evaluation is observation; there are some studies which use self evaluation for practice (42). By the way, use of self evaluation is important limitation of this study. In addition using limited questions for evaluation of knowledge objectively may be another limitation of current research. 

Finally as the attitude and practice are highly affected by many other factors such as pharmacists remuneration models (43), further studies should explore other underlying factors on attitude and practice of pharmacists. 
